# Selenium Nanomaterials to Combat Antimicrobial Resistance

**DOI:** 10.3390/molecules26123611

**Published:** 2021-06-12

**Authors:** Linh B. Truong, David Medina-Cruz, Ebrahim Mostafavi, Navid Rabiee

**Affiliations:** 1 Department of Chemical Engineering, Northeastern University, Boston, MA 02115, USA; truong.li@northeastern.edu (L.B.T.); medina.dav@northeastern.edu (D.M.-C.); 2 Stanford Cardiovascular Institute, Stanford University School of Medicine, Stanford, CA 94305, USA; 3 Department of Medicine, Stanford University School of Medicine, Stanford, CA 94305, USA; 4 Department of Chemistry, Sharif University of Technology, Tehran 11155-3516, Iran

**Keywords:** selenium nanomaterials (SeNMs), antimicrobial, green nanotechnology, nanoparticles (NPs)

## Abstract

The rise of antimicrobial resistance to antibiotics (AMR) as a healthcare crisis has led to a tremendous social and economic impact, whose damage poses a significant threat to future generations. Current treatments either are less effective or result in further acquired resistance. At the same time, several new antimicrobial discovery approaches are expensive, slow, and relatively poorly equipped for translation into the clinical world. Therefore, the use of nanomaterials is presented as a suitable solution. In particular, this review discusses selenium nanoparticles (SeNPs) as one of the most promising therapeutic agents based in the nanoscale to treat infections effectively. This work summarizes the latest advances in the synthesis of SeNPs and their progress as antimicrobial agents using traditional and biogenic approaches. While physiochemical methods produce consistent nanostructures, along with shortened processing procedures and potential for functionalization of designs, green or biogenic synthesis represents a quick, inexpensive, efficient, and eco-friendly approach with more promise for tunability and versatility. In the end, the clinical translation of SeNPs faces various obstacles, including uncertain in vivo safety profiles and mechanisms of action and unclear regulatory frameworks. Nonetheless, the promise possessed by these metalloid nanostructures, along with other nanoparticles in treating bacterial infections and slowing down the AMR crisis, are worth exploring.

## 1. Introduction

Bacterial infections had killed more people worldwide than any other causes before the 1920s, when the first class of antibiotic-based treatment for bacterial infections, penicillin, was introduced. Following a successful implementation, the golden age of antibiotics started with the discovery of a wide variety of antimicrobial agents, including, but not limited to, streptomycin and vancomycin [1]. Nonetheless, decades later, lower respiratory infections were established as the fourth leading cause of death globally, among the prevalence of other bacterial pathologies [2]. The reason behind this persistence was an accelerated rise of antimicrobial resistance to antibiotics, also known as AMR. This evolutionary and induced behavior provides bacteria with the ability to fight the drugs that are supposed to kill them. Decades after, humanity lives in the post-antibiotic era, and the AMR crisis has shortly become one of the biggest challenges to face. 

AMR provides bacteria with the ability to develop various mechanisms that protect them from the effects of the antibiotics. Although not a new problem, rather a feature of bacteria that has always been present, the AMR crisis has been amplified through the severe overuse and misuse of current antibiotics in healthcare, agriculture and livestock, among other settings [3]. Unfortunately, the traditional discovery of antibiotics-based treatments cannot keep up with the evolution of bacteria, which leads to an uncertain future. By 2050, AMR will lead to more than 10 million deaths worldwide, with a cumulatively financial cost of USD 100 trillion [4]. This crisis requires urgent attention and a more innovative approach. 

Current treatments to bacterial infections consist mainly of combining traditional classes of antibiotics and new drugs whose use is extremely limited amid the fear of AMR surges. Simultaneously, only nine new chemically synthesized antibiotic agents have been approved from 1998 to 2003, of which only two exhibited new mechanisms of action [5]. Therefore, there have been multiple efforts to tackle the current issues outside the scope of antibiotics with alternative approaches such as antimicrobial peptides (AMPs), phage therapy (PT), and nanomaterials (NMs). 

AMPs are short strings of amino acids coded naturally in various organisms’ genetic materials to fight bacterial infections through membrane disruption, among other mechanisms [6]. However, while effective, AMPs discovery is time-consuming, expensive, and difficult to process [7]. Additionally, there is an increasing concern over using synthetic copies of AMPs, leading to a faster resistance employing a common mechanism that bacteria have to avoid these new drugs [8,9]. On the other hand, PT employs viruses to lyse bacterial cells by targeting specific receptors that can bind to a certain range of bacteria. Although PT is unlikely to lead to resistance due to its specificity of the target and its critical importance to the bacteria [10], some cases of resistance in laboratory settings have been described [11]. However, just as bacteria can evolve to show resistance, viruses can evolve to overcome it. Nevertheless, PT cannot function as a broad-spectrum treatment, and its clinical evidence and regulatory framework are limited [12]. While both AMPs and PT hold some promise, they present challenges to the fast discovery and adaptation of new infectious treatment that the current crisis demands.

**Figure 1:**
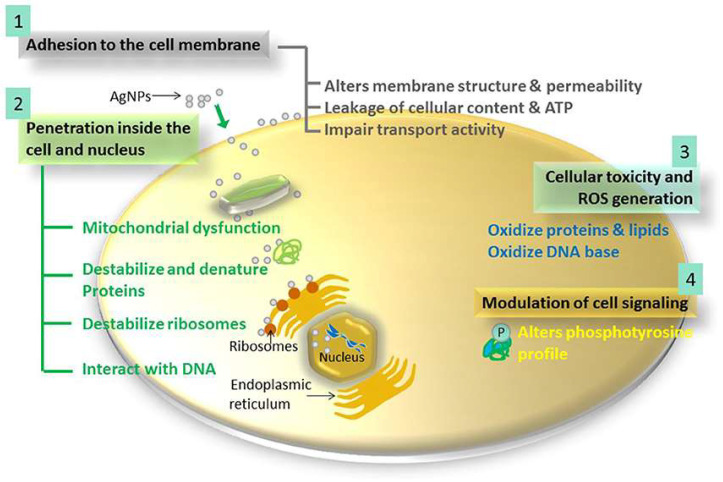
Schematic of the four main mechanistic actions of AgNPs against bacterial cells, resulting in cell death [16]. Reprinted with permission.

On the other hand, nanotechnology presents an alternative approach to traditional antibiotics and offers new drugs that might overcome some noted limitations [13]. NMs can both be used as a carrier of other therapeutic agents or as therapeutic agents themselves. As such, nanoparticles (NPs) composed of polymers and lipids have been investigated as drug vehicles over the last few decades. Evidence pointed to an increase in safety of the delivered molecules and effectiveness against microbial infections [14]. Another type of NMs, metallic NPs (MNPs), has also been widely investigated in laboratory settings due to their inherent antimicrobial properties related to the features of their metallic constituents [15]. Mechanism of actions for MNPs include: (a) attraction to bacterial cell wall due to opposite surface charges; (b) destabilization of the membrane; (c) generation of reactive oxygen species (ROS); (d) metal ion release; and (e) alternation of the signaling pathway, as demonstrated in Figure 1 [16]. The most common MNPs elements, currently under investigation, include silver (Ag), gold (Au), copper (Cu), Zinc (Zn), and all of their oxide derivatives [17,18,19,20]. Nevertheless, while commonly studied structures such as silver NPs (AgNPs) can certainly be effective against bacteria, even resistant strains, they also bring some drawbacks to the table, mainly regarding their toxicity and further bacterial mutations [21]. As such, some studies report the development of bacterial resistance against AgNPs, which have been extensively used for medical purposes [22], while they are also able to induce horizontal transfer of antibiotic resistance genes [23]. 

Due to the above, nanotechnology would benefit from exploring different alternatives within the periodic table. One of the most promising options comes from the use of metalloid NPs, which include those made of selenium (Se) and tellurium (Te). In this review, the main focus will be around selenium nanoparticles (SeNPs), those made of the chalcogen element, a scarce substance in biological systems, yet essential for many organisms. Importantly, SeNPs can prevent further environmental antibiotic resistance genes transfer when used in the nanoscale, rendering efficient antimicrobial features without causing difficulty in the scale-up harvest. Moreover, SeNPs exhibit other biomedical applications, including anticancer and antioxidant properties [24], while showing enhanced efficacy, bioavailability, and biological activity [25]. Additionally, and important in the current situation, SeNPs can potentially offer a beneficial role as antiviral agents. Due to their antimicrobial activities, SeNPs can exhibit an enormous potential in antiviral applications, both by detecting viral illness at an early stage and inducing inhibition of viral infections through targeted drug delivery [26]. These attractive qualities incentivize researchers to apply SeNPs into the antimicrobial field, potentially presenting more effective treatments of current common and resistant bacteria strains, as well as other microbial pathogens, without the problems associated with more traditional NMs. 

Current strategies for producing most NMs, including SeNPs, are mainly divided into traditional and green synthesis methods [27,28]. Traditionally, SeNPs can be produced by employing physical, chemical and combination methods that employ both platforms. While physical synthesis utilizes either heat or force to manipulate the size of the NPs produced from its corresponding ions or bulk material form, chemical platforms rely on the reduction of Se ions into the elemental form through reducing and stabilizing agents. Among physical methods, thermal deposition, mechanical or ball milling, lithographic approaches, laser ablation, and sputtering are widely employed in the production of NPs. In contrast, techniques such as sol-gel methods, co-precipitation, or chemical reductions are often found among the chemical synthesis approaches [29]. Despite the straightforwardness and reproducibility of these methodologies, they often suffer from drawbacks, such as the production of toxic by-products and the use of harsh reaction conditions during processing. 

On the other hand, to overcome such limitations, green synthesis of NPs offers biological organisms and biomolecules a way to reduce waste materials and capping agents to generate the NPs in a cost-effective, environmentally-friendly, and safe production environment [24]. The outcome is NPs with similar properties and features as those traditionally produced but often showing higher biocompatibility and biodegradability, with no need for further functionalization with other molecules or stabilizing agents. 

Therefore, this review article will detail the most recent advances in both traditional and green synthesis of SeNPs in the fight against AMR bacteria, discussing the advantages and drawbacks of both approaches. First, the traditional synthesis of SeNPs is discussed, including those NPs made by physiochemical methods, including, but not limited to, precipitation, chemical reduction in the presence of surfactants, and pulsed laser ablation, among others. These methods produce nanomaterials in a straightforward and relatively reproducible fashion but necessitate further functionalization, incur toxic products, and require specialized instruments. Secondly, green nanotechnology-based methods are presented, including the use of natural raw materials such as bacteria, fungal and plant extracts, or naturally derived biomolecules to produce SeNPs. These methods can greatly overcome some of the limitations of their traditionally synthesized counterparts and greatly enhance the biocompatibility and biomedical properties of the SeNPs. Nonetheless, the scalability, reproducibility, and safety of biogenic SeNPs present some obstacles toward their clinical applicability. Finally, the current state and future perspective of SeNP applications against AMR bacteria are examined to explore the potential of these NPs as key assets in the future of healthcare. 

## 2. Traditional Synthesis of SeNPs for Fighting AMR

Traditional synthesis of NMs, including chemical, physical, and physicochemical techniques, presents the most established and well-known array of processes for the straightforward, reproducible, and homogeneous nanostructure synthesis [30]. These methodologies have been successfully implemented in the production of SeNPs, offering suitable approaches that can have multiple biomedical applications, especially those related to AMR treatment. 

Most of these traditional synthesis methods can be related to chemical methods reported for the synthesis of SeNPs that reduce Se salts (mainly sodium selenite, Na_2_SeO_3_) using various reducing agents, including surfactants or biocompatible chemicals to obtain stabilized colloidal suspensions of SeNPs of various shapes and sizes [31]. Chemical reduction methods help maintain better uniformity of the NPs [32]. In this section, several chemical methods to produce SeNPs to combat antibiotic-resistant bacterial infections are explored (Table 1).

**Figure 2:**
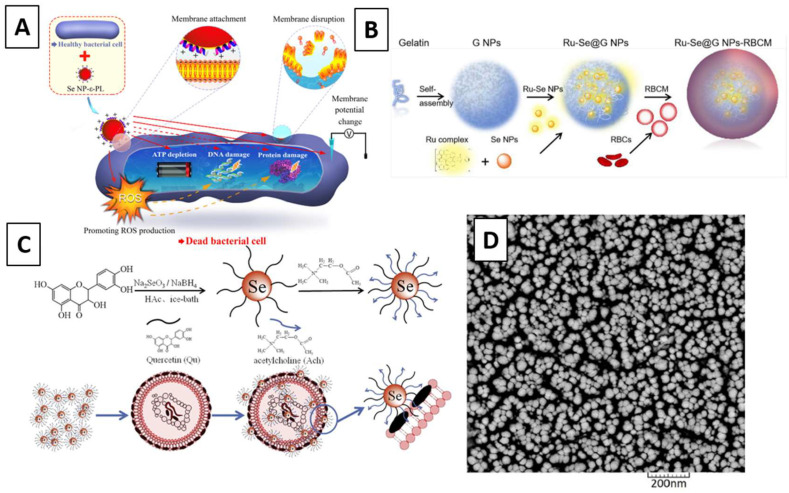
Schematic of the hypothesized antibacterial mechanism of SeNPs can attach to the bacterial cell membrane through electrostatic interactions to damage the bacterial cell by promoting ROS production, depleting ATP, changing membrane potential, and disrupting the membrane (indicated by red solid arrows). (**A**) Se NP-ε-PL has the potential to induce DNA damage and protein damage as well (indicated by red dashed arrows [35]; (**B**) scheme of the synthesis process for Ru-complex-functionalized SeNPs loaded into gelatin NPs [37]; (**C**) synthesis process for a synergistic nanocomposite produced by the conjugating of quercetin (Qu) and acetylcholine (Ach) to the surface of SeNPs [39]; (**D**) scanning electron microscopy (SEM) images of chemically synthesized SeNPs [41]. Reprinted with permission.

One interesting article was employed by Huang et al. through the chemical reduction of selenium dioxide (SeO_2_) with sodium thiosulfate (Na_2_S_2_O_3_) as the reducing agent and polyvinyl alcohol (PVA) as the stabilizer. Spherical SeNPs were fabricated and tested for their antibacterial activity, showing a strong size-dependent activity with maximal growth inhibition and killing effect toward Methicillin-susceptible *Staphylococcus aureus* (MSSA) and Methicillin-resistant *Staphylococcus aureus* (MRSA). A multi-modal mechanism of action dependent on the NPs sizes was reported, including depleting internal adenosine triphosphate (ATP), inducing ROS production, and disrupting membrane potential. The authors also reported that the size was a facile yet critical and previously underappreciated parameter tailored for maximal antimicrobial efficacy [33]. In another example, 30–70 nm SeNPs were produced utilizing the chemical reduction of Na_2_SeO_3_ with L-ascorbic acid and PVA. Once produced, the SeNPs were used as a coating through surface-induced nucleation-deposition on titanium implants. The antimicrobial activity against drug-resistant bacteria including MRSA and Methicillin-resistant *Staphylococcus epidermidis* (MRSE) was investigated in vitro and within an infected femur model in rats. The SeNPs coatings strongly inhibited biofilm formation on the implants and reduced the number of viable bacteria in the surrounding tissue following inoculation of implants with biofilm-forming doses of bacteria at concentrations as low as 0.5 ppm [34]. Similarly, the same chemical processing was conducted to produce SeNPs coated with the antimicrobial polypeptide ε-poly-l-lysine, which exhibited significantly greater antibacterial activity against drug-resistant strains than their components, SeNPs and ε-PL. Their mechanisms of action are proposed in Figure 2A. The authors further demonstrated that the SeNPs did not readily induce resistance in *Escherichia coli* or *Staphylococcus aureus* [35]. Furthermore, SeNPs with sizes between 55–500 nm were produced by chemical reduction of Na_2_SeO_3_ by mercaptopropionic acid in the presence of chitosan and used as antibacterial agents in combination with a porous antibacterial collagenous scaffold. Even at concentrations as low as 5 ppm of SeNPs, modified polymeric scaffolds showed considerable inhibition activity toward Gram-positive bacterial strains such as MRSA in a dose-dependent manner [36].

In a different example, SeNPs were produced by reduction of Na_2_SeO_3_ using glutathione in an aqueous solution, functionalized with Δ-[Ru(phen)_2_(tip)](ClO_4_)_2_·2H_2_O and then loaded into gelatin NPs, which were coated with a red blood cell membrane comprising a typical lipid bilayer membrane to construct a biomimetic multifunctional antibacterial nanosystem, as demonstrated in Figure 2B. *In vitro* experiments demonstrated that the nanosystem could escape the phagocytosis of macrophages, effectively reduce the hemolytic activity of MRSA secreted exotoxins, and deliver the NPs to destroy bacteria cell structure. The ability to monitor infection treatment progress and effectively promote wound healing of bacterial infection was also proved by in vivo MRSA-infected mice models [37]. Moreover, SeNPs were synthesized by chemical reduction of Na_2_SeO_3_ in ascorbic acid and then conjugated with chitosan and mupirocin. In vitro studies were performed by evaluating the antibacterial activity and toxicity on the L929 mouse fibroblast cell line. The in vivo study was conducted on a rat diabetic wound infection model infected by mupirocin-methicillin-resistant *Staphylococcus aureus* (MMRSA). The wounds were treated with the SeNP-based nanohybrid system two times/day for 21 days. This therapeutic effect was evaluated by monitoring wound contraction and histopathological changes [38].

Furthermore, Huang et al. synthesized a synergistic nanocomposite by conjugating quercetin and acetylcholine to the surface of SeNPs prepared by chemical reduction of Na_2_SeO_3_. The schematic of the process is illustrated in Figure 2C. Quercetin has been reported to exhibit a wide range of biological activities related to its antibacterial activity and acetylcholine as a neurotransmitter. The authors demonstrated antibacterial efficiency against multidrug-resistant superbugs (MDRs), such as MRSA, at a low dose. The studies showed that the NPs attach to the bacterial cell wall, causing irreversible damage to the membrane, and thereby achieving a remarkable synergistic antibacterial effect to inhibit MRSA. The findings suggest that quercetin and acetylcholine’s synergistic properties enhance the antibacterial activity of SeNPs [39]. Alternatively, Cihalova et al. reported a strong inhibition effect of SeNPs in complexes with conventional antibiotics. Using an impedance method, a higher disruption of biofilms was observed after applying antibiotic complexes with SeNPs compared with those exposed to antibiotics without SeNPs. The biofilm formation was inhibited up to 94% ± 4% for MRSA after applying the NPs compared with bacteria without antibacterial compounds, whereas antibiotics without SeNPs inhibited MRSA only up to 16% ± 2%. The obtained results provide a basis for the use of SeNPs as a tool for treating bacterial infections, which can be complicated because of the increasing resistance of bacteria to conventional antibiotics [40]. Alternatively, a nanohybrid system integrating SeNPs with lysozyme was produced based on the potential of a good synergistic effect following a facile wet chemical method to produce the SeNPs, shown in Figure 2D, and a fixed concentration of lysozyme was added. Antibacterial tests were done against *S. aureus* and *E. coli*, showing that the SeNPs play an important role in inhibiting bacterial growth at a very low concentration of protein. Additionally, individual NPs were shown to efficiently reduce bacterial growth at low concentrations concerning very high concentrations of lysozyme [41].

Employing a physical method that was also environmentally friendly, pulsed laser ablation (PLAL) was used to produce SeNPs in liquids. The protocol was optimized to produce a large amount of SeNPs within 10 min of irradiation. The SeNPs were tested on MDR *E. coli* and MRSA, showing a cell death mechanism related to ROS production while remaining cytocompatible with healthy human cells [42]. In another study, a blend of polyvinyl alcohol/chitosan doped by SeNPs was prepared. The SeNPs were doped and distributed in the PVA/chitosan blend via laser ablation using a selenium plate immersed and ablated in the PVA/chitosan solution. Then, the PVA/chitosan/SeNPs nanocomposite films were created using solution casting and dried in a furnace at 45 °C for three days. The antibacterial activity was assessed against *E. coli*, *Pseudomonas aeruginosa*, *S. aureus,* and *Bacillus subtilis*. The activity index showed an increase in the diameter zone with an increasing concentration of SeNP content, leading to increased antibacterial activity compared with the pure PVA/chitosan blend [43]. 

Once examples of traditional synthesis of SeNPs to combat AMR bacteria have been introduced, it is clear that physical approaches are currently not as common as the chemical ones. However, it is important to note that physical synthesis would present some advantages, including the lack of contamination from other chemical and biological molecules, thus maintaining Se integrity and purity [44]. Physical approaches also often do not require separation and purification steps, saving valuable time and materials. However, these methods sometimes require high energy consumption and expensive equipment (such as laser ablations or gamma irradiation) whose maintenance is costly enough [45]. Combined with its relative energy inefficiency, scale-up processes become the main obstacle for the physical synthesis approach. 

In contrast, chemical reduction has become the most popular method for the synthesis of SeNPs. The procedures have been widely established, including lists of reducing agents that continue to expand. Most of these reacting chemicals are cheap and available, allowing researchers to perform experiments [46]. Traditional approaches also allow for the smaller size of NPs due to their bottom-up synthesis nature, which is desirable due to the positive correlation between smaller NPs and better antimicrobial properties. Another important advantage includes the ready ability to design and functionalize SeNPs. Whether concerning shape, size, structures, or surface modifications, the chemical approach to SeNP synthesis provides researchers with the ability to be creative without deviating from an established procedure. Nevertheless, as stated, chemical approaches can require multiple separations and purification steps. While it could be scaled up, this process can become inefficient, hazardous, and expensive. 

## 3. Green-Synthesized SeNPs

**Figure 3:**
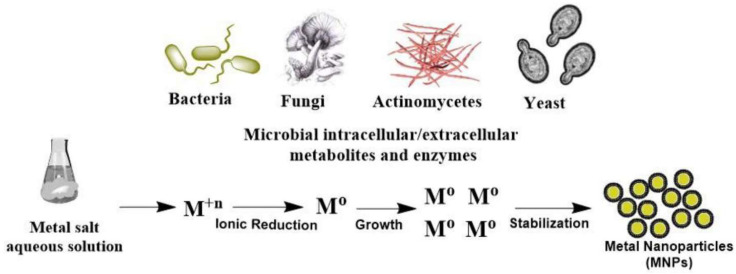
The overall mechanism of green synthesis of metal nanoparticles through natural reduction of metallic ions by microbial enzymes [49]. Reprinted with permission.

**Figure 4:**
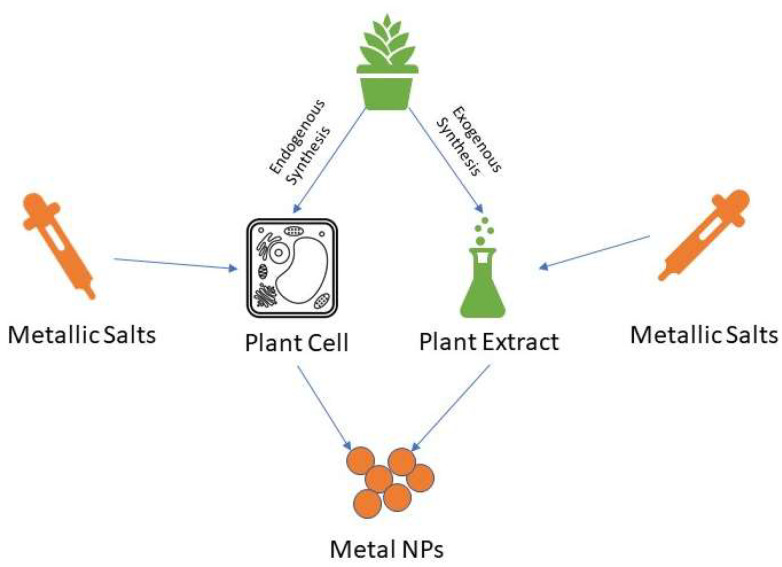
Overall procedural diagram for both endogenous and exogenous synthesis of metal NPs using plants. When working with plants as raw materials for synthesis of NPs, both cells (when using entire living organisms) and extracts (when using parts of the plants) must be considered as factors for the growth of the NPs, producing different sizes, shapes, and other properties that will impact the biomedical applications of the produced nanostructures.

Green synthesis of SeNPs relies on both the use of living organisms and associated biomolecules to reduce Se ions into SeNPs. Multiple organisms can be used as biofactories, with bacteria, fungi, and plant extracts as the most common approaches that allow producing valuable NMs at ambient conditions [47]. These organisms contain several reductase enzymes, whose function are to detoxify lethal selenite ions into elemental selenium [48,49]. The general mechanism of green synthesis of metal NMs is presented in Figure 3, with the specific focus on different pathways of plant-mediated synthesis presented in Figure 4. These biosynthesized SeNPs maintain the properties exhibited by SeNPs produced physiochemically yet can potentially add more desired qualities due to the biologic environment they were made from. 

Additionally, it is important to mention that nature can provide an invaluable source of inspiration for designing novel NMs with potent activity against bacterial-resistant pathogens by successfully integrating bioinspired approaches along with material design in the field of nanotechnology. The potential of green production of antimicrobial NPs concerning different biological origin and different elemental materials prove to be a worthwhile effort that is being examined meticulously [50]. Once generated, these nanomaterials can be used beyond their intended scope and can support other biomedical applications and uses [51]. 

### 3.1. Bacteria-Mediated Synthesis of SeNPs

Bacteria are the oldest living organisms to inhabit the planet, and after millennia of adaptations to the environment and evolution, they have conquered every corner of Earth. As such, they have developed the ability to tolerate some of the harshest environments, including those with a high presence of metal reservoirs, such as Se-based environments. To survive those ecosystems, these organisms have learned how to cope with these metals transforming ions into elemental forms. Therefore, bacteria are frequently considered natural nano-biofactories due to their fast growth rate, minimal maintenance, and economical processing. Reports of NPs synthesis by bacteria, including, but not limited to AgNPs and AuNPs, are widely present in literature [52].

In terms of SeNP bacterial production, some reports have been found in the literature [53]. For its antimicrobial applicability, the work of Srivastava et al. highlighted the use of *Ralstonia eutropha*, a Gram-negative bacterium, to produce SeNPs by inoculation and exposure to 1.5 mM of Na_2_SeO_3_ solution for 48 h. A color change to red was observed, and UV-Visible Spectrometry (UV-Vis) confirmed the presence of SeNPs in solution with a size distribution of 40–120 nm. The study demonstrated that biogenic SeNPs treatment was comparable to ampicillin, as demonstrated by their MIC values against multiple pathogenic species. Interestingly, biogenic SeNPs MIC value against *S. aureus* was more than half of ampicillin [47]. Building from Srivastava’s work, numerous studies were done to examine the potential of biogenic SeNPs as an antibacterial agent. For instance, *Bacillus mycoides* and *Stenotrophomonas maltophilia* were used to synthesize SeNPs, and their properties were compared with chemically synthesized SeNPs. The authors found that biogenic SeNPs showed biological macromolecules that were not apparent in their chemical synthesis. Additionally, biogenic SeNPs were slightly larger, but their antimicrobial properties against *P. aeruginosa* were comparable to chemically synthesized SeNPs [54].

To further optimize the bacterial-mediated SeNPs synthesis, Shoeibi et al. utilized *Enterococcus faecalis* as biofactories. Several concentrations of Na_2_SeO_3_ were tested. Interestingly, higher selenite ion concentrations did not lead to better quantity or efficiency of produced SeNPs. The authors indicated that the optimal concentration of ions should be 0.19 mM and hypothesized that higher selenite concentration would kill the bacteria, thus eliminating the mechanism to reduce them [55]. In the same study, the antimicrobial property of SeNPs was confirmed on *S. aureus* using disk diffusion assay, with a zone of inhibition (ZOI) size of 8 mm. More recently, Ramya et al. synthesized SeNPs using *Streptomyces* sp. This Gram-positive bacterium rich in bioactive metabolites, including those that act as a natural antibiotic, was proposed as a suitable candidate for biogenic SeNPs synthesis. Antimicrobial assays were performed with stand-alone SeNPs and in combination with common antibiotics. It was found that the MIC values against several strains of pathogenic bacteria were in the range of 80–120 ug/mL. Significantly, the study provided evidence of a synergistic effect when combined with common antibiotics, showing promise to a more potent treatment [56].

**Figure 5:**
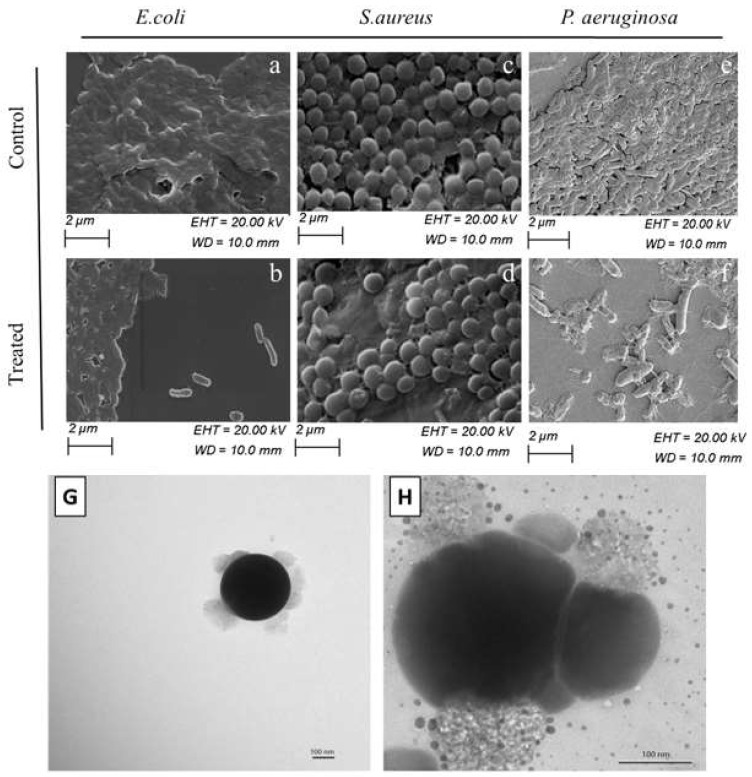
SEM images of *E. coli*, *S. aureus* and *P. aeruginosa* with LA-SeNPs at MIC concentration (**b**,**d**,**f**) and without LA-SeNPs (**a**,**c**,**e**) [57]; transmission electron microscopy (TEM) images showing the nanoparticle coating. Natural coating surrounding the spherical structure of nanoparticles made by Staphylococcus aureus (**G**) and *Escherichia coli* (**H**) [59]. Reprinted with permission.

Biogenic SeNPs were shown to perform better in killing resistant bacteria strains than gentamycin, a common antibiotic. In the study, *Lactobacillus acidophilus* was used to synthesize SeNPs. The MIC values for the SeNPs treatment were comparable with gentamycin against common pathogens, yet significantly lower against more resistant strains such as *P. aeruginosa* and *Klebsiella pneumoniae* at 4–6 ug/mL compared to 120–180 ug/mL. The study also indicated evidence in favor of ROS as part of the SeNPs mechanism of action. The SeNPs exhibited a concentration-dependent effect on deterring biofilm formation, potentially eliminating another pathway to infections, as demonstrated in Figure 5a–f [57]. The multifunctionality of biogenic SeNPs as antimicrobial agents and biofilm eradication treatments was confirmed in other studies as well [58]. Medina Cruz et al. suggested an approach for increasing SeNPs treatment effectiveness by tuning the treatment to that of the targeted pathogen. SeNPs were synthesized by pathogenic organisms, including *E. coli* (shown in Figure 5H), *P. aeruginosa, MRSA* (shown in Figure 5G), and *Staphylococcus epidermis*. These SeNPs were then treated against the organisms they were made from, showing a significant increase in potency compared with cross applications against other bacteria. Additionally, these SeNPs showed no or limited cytotoxicity against human dermal fibroblast (HDF), alleviating some of the safety concerns associated with the method of production [59].

Few studies have attempted to demonstrate the improved efficacy of bacterially synthesized SeNPs over the traditional mode of production. Piacenza et al. compared the antimicrobial effect between chemically reduced SeNPs using l-cysteine with biologically produced SeNPs using the Gram-positive bacterium *B. mycoides*. The study confirmed organic elements on biogenic SeNPs, which exhibited superior biofilm prevention, biofilm eradication, and bacterial inhibition ability. Interestingly, biogenic SeNPs produced after 6 h of exposure performed better than those produced after 24 h. This phenomenon was hypothesized to be due to the correlation between size and exposure time to the NPs, combined with the correlation between the size and activities of the NPs [60]. 

### 3.2. Fungi-Mediated Synthesis of SeNPs

The use of fungi to synthesize NPs has long been investigated for its potential in agriculture, specifically disease management. Fungal organisms can hold and secrete more enzymes than smaller bacterial species, giving them more catalytic power to convert metallic ions into metallic NPs [61]. Earlier work on fungi-mediated SeNP synthesis was carried out by Hariharan et al. Strain yeast *Saccharomyces cerevisiae* was cultured and exposed to selenite salt at different concentrations for 24 h. The recovered product was analyzed under UV-Vis and X-ray diffraction analysis (XRD) to confirm SeNPs presence. Interestingly, the optimal concentration of selenite salt was at 2 mM, consistent with the optimal condition for bacterial biosynthesis. Antimicrobial assays indicated that the resulting SeNPs were most effective against *E. coli* and *S. aureus*, with MIC of 31.25 ug/mL [62].

The synergistic properties between fungal-assisted synthesized SeNPs and antibiotics were suggested. Specifically, *Penicillium chrysogenum* filtrate was used to biosynthesize SeNPs in the presence of gamma rays, followed by the incorporation of gentamycin—a common antibiotic. Significantly, the result indicated that the combination of SeNP and gentamycinhad greater efficacy than each one of them separated, giving MIC values of 0.245–3.95 ug/mL against all tested microorganisms, including both Gram-positive and Gram-negative bacteria. Characterization also indicated that the average particles’ size was 33.84 nm, giving evidence that gamma irradiation treatment can be used in combination with green synthesis to control the size [63]. A cell-free filtrate of *Penicillium corylophilum* was studied for its SeNPs synthesis and subsequent antimicrobial activities. The study also reported that antimicrobial properties were more potent in Gram-positive bacterial strains than Gram-negative species. Interestingly, SeNPs were also shown to exhibit larvicidal activity, opening the door to a new field of applications [64]. An extensive study focusing on multidrug-resistant bacteria was carried out, in which the treatment was SeNPs produced from *Aspergillus oryzae* extract in the presence of gamma radiation. The resulting NPs were tested against a panel of Gram-positive and Gram-negative MDR pathogens, including resistant strains of *Staphylococcus*, *Enterococcus*, *Enterobacter*, and *Pseudomonas* families. The exposure to the SeNPs exhibited consistent and superior results compared with amoxicillin [65].

### 3.3. Plant-Mediated Synthesis of SeNPs

While the employment of bacterial and fungal organisms can lead to some safety concerns due to the hosts’ biomolecules presence, plants give a new alternative. In addition, plants offer both endogenous and exogenous production of NPs in the form of their extracts, where residue cells and secreted enzymatic chemicals act as reducing agents for metallic ions, shown in Figure 4 [66]. Therefore, SeNPs production using plants are eco-friendly, inexpensive, versatile, and scalable. The availability of materials encouraged research to grow for the plant-assisted synthesis of SeNPs [67]. 

For instance, *Leucas lavandulifolia*, a common lavender leaf in Africa and east Asia, was utilized for biosynthesis in its leaf and stem extract. The presence of SeNPs was detected and confirmed. The research proposed that biomolecules within the extracts, such as its polyphenol and heterocyclic components, were responsible for reducing Se ions. Antimicrobial properties were observed through ZOI parameters, ranging from 13–16 mm in all tested bacteria. However, the effectiveness of the extract was not as high as a standard antibiotic in the study [68]. Many antimicrobial properties were also observed in SeNPs synthesized by *Withania somnifera*, with ZOI in the range of 12–20 mm, with its highest effectiveness against *S. aureus*. The authors also suggested that the resulting SeNPs also exhibited antioxidant and photocatalytic activities, which enhanced their multifaceted pharmaceutical properties [69]. 

Even the most common plants could be used to produce SeNPs, as demonstrated by the use of ginger extract. The synthesis indicated that the optimal condition was at 10 mM of Na_2_SeO_3_, highlighting plant extract potential to handle higher exposure to Se ions. Gram-negative bacteria such as *Proteus* sp. and *Serratia* sp. were the most susceptible to the NPs exposure. In addition to the disk diffusion assay, the growth kinetics of bacteria was also studied in the presence of SeNPs, with the result indicating that the exposed bacterial growth did not achieve its standard exponential kinetics [70]. With the ability to handle higher exposure to Se ions, plant-mediated SeNPs synthesis can occur in a shorter time. Rapid synthesis of SeNPs using *Azadirachta indica* was performed within 5–10 min, and the resulting SeNPs could be controlled by varying the reduction time. These nanostructures exhibited comparable antimicrobial to Ampicillin while remained cytocompatible to human L929 fibroblast cell line [71].

The resulting SeNPs produced from plant extracts were proven to be compatible and safe to human cell lines while remaining potent to pathogenic bacteria. For instance, the biomass of *Spirulina platensis* was used to synthesize SeNPs, and its antimicrobial properties were demonstrated against clinical isolates of *K. pneumoniae* with MIC values of 25–250 ug/mL. A cytotoxicity study was performed against liver and kidney cells, showing no cytotoxicity effects on both cell lines [72]. This finding was consistent with previous studies, reassuring the safety of plant-mediated NMs [73].

Overall, multiple species of bacteria, fungi, and plants have been shown to successfully synthesize SeNPs with different sizes and properties, as detailed in Table 2. A common trend that was observed was that organic elements were found on the SeNPs, suggesting attached biomolecules from the host. Antimicrobial properties were found against both Gram-positive and Gram-negative pathogenic bacteria, but their bactericidal strength depended on different synthesis methods. In addition to their comparable antimicrobial effects, the green synthesis processes generate NPs in a more environmentally friendly, versatile, energy-efficient, and economical approach than traditional methods [74]. While it is not well understood as to why the effects of SeNPs vary based on their host biofactories, biogenic SeNPs promise a solution for resistant strains of pathogens while providing alternative treatment to alleviate common bacteria resistance development. 

## 4. Conclusion and Future Prospect

Overall, this work details the current advances and comparisons of different SeNPs synthesized by two different pathways and their subsequent applications as antimicrobial treatments, focusing on the treatment and applicability in the fight against AMR. The different articles show that SeNPs are powerful antibacterial agents, yet safer to human cells than bulk and ionic forms of the chalcogen element. In their use as antimicrobial agents, SeNPs have shown promise as an alternative treatment of bacterial infections or as combination agents, reducing the loads that current antibiotics carry and their use. As such, lesser exposure to standard antibiotics would help prevent rapid AMR, an important task given that the rate of resistance currently outpaces the rate of new drug discoveries.

Traditional methods of synthesis of SeNPs present notable advantages, including the lack of after-synthesis processing in physical methods, easy and versatile functionalization with different biocompatible agents, and realistic scalability when needed. Nevertheless, these methodologies need a more efficient and ecologically responsible approach to reduce the environmental footprint associated with them and to reduce the release of unwanted and toxic by-products that suppose a harm to both the environment and society. Therefore, green synthesis of NPs has become more popular due to its comparable potency, lowered expenses, scalability, and sustainability. Additionally, when employed in SeNPs, green nanotechnology-based approaches found a perfect candidate due to the inherent biocompatibility and bioavailability of the metalloid. However, biogenic SeNPs face challenges, including the lack of reproducibility in the synthesis protocols, the homogeneity of the produced particles (for instance, in features such as size or shape), and the overall poorer understanding of the mechanism of actions, which limit their preclinical studies and clinical introduction. In addition, a more detailed safety profile for traditional and biogenic SeNPs presents a priority for expediting their therapeutic translation. Furthermore, a more robust processing, manufacturing, and regulatory infrastructure need to be established, such that purification and characterization of SeNPs are readily available. 

Importantly, how the use of SeNPs impacts their potential toxicity in living organisms must be considered. Although the nanoscale form of Se provides a significant reduction in toxicity compared with inorganic and organic selenium compounds, the NPs still confer some toxicity [78]. When shown, the toxicity of SeNPs is almost exclusively associated with the induction of oxidative stress in cells [79]. For instance, the toxicity of SeNPs measured by 24 h half maximal inhibitory concentration IC_50_ values ranged from 1.4 to >100 mg Se/L, depending on surface functionalization and was not caused by ionic Se that might be degraded from the NPs core. At subtoxic concentrations, all SeNPs were taken up by immortalized human keratinocytes (HaCaT), epithelial (TR146) and colorectal adenocarcinoma (CaCo-2) cells, induced oxidative stress response, and demonstrated genotoxicity [80]. However, most of the ranges of concentrations that provide antimicrobial effects have not demonstrated impact on the proliferation of healthy tissue in both in vitro and in vivo studies [81]. As such, some studies concluded that supranutritional levels of SeNPs had no obvious toxic effects in rats and could be used as potential antimicrobial agents within a defined concentration range [82]. Additionally, it is important to mention that biologically produced SeNPs are more stable and biocompatible than traditionally produced NPs, owing to the natural coating of biomolecules. As such, biogenic SeNPs were reported to be 26-fold less toxic than SeO_2_ and traditionally-synthesized SeNPs [83], offering a clear advantage in terms of coping with potential toxicity. As more studies are conducted, a greater confidence in the safety profiles of SeNPs in living systems can be obtained, opening the door for potential clinical usage.

The next wave of research should also explore the detailed biogenic production mechanism and better understand (a) the location of reduction, (b) the effect on host biofactories, and (c) the exporting pathways. Once these are understood, the produced SeNPs would be better than those produced by traditional synthesis approaches. Once done, a more detailed comparison and evaluation of features would be possible. Eventually, such a library of research would allow the safe use of SeNPs as efficient antimicrobial agents, allowing for a successful screening of available particles in terms of their therapeutic effect and enabling an exceptional treatment against pathogenic bacteria that might significantly improve the outcomes of the AMR crisis that society is going to face in the near future.

## Figures and Tables

**Table 1:** Recent advances in the physiochemical synthesis of SeNPs toward antimicrobial applications.

Method	Precursor	Reducing Agent	Stabilizing Agent	NP Size (nm)	Tested Bacteria	Antibacterial Parameters	Ref.
Chemical reduction	SeO_2_	Na_2_S_2_O_3_	PVA	43–205	MRSA	MIC: 12 ± 2 μg/mL	[33]
	Na_2_SeO_3_	C_6_H_8_O_6_	PVA	50–200	MRSA	GC inhibition at 0.5 ppm	[34]
					MRSE	GC inhibition at 0.5 ppm	
	SeO_2_	Na_2_S_2_O_3_	PVA/ε-PL	80	MRSA MDR *K.Pneumonia*	MIC: 8.6 ± 4.2 μg/mL MIC: 26.2 ± 0.4 μg/mL	[35]
	Na_2_SeO_3_	HSCH_2_CH_2_CO_2_H	>CMC	50–300	MRSA	GC inhibition at 5 ppm	[36]
	Na_2_SeO_3_	Glutathione	BSA	20–152	MRSA	GC inhibition at 25 μg/mL	[37]
	Na_2_SeO_3_	C_6_H_8_O_6_	N/A	61 ± 7	MRSA	MIC: 32 μg/mL	[38]
	Na_2_SeO_3_	Acetylcholine chloride	Quercetin	80 ± 10 (Qu@SeNPs)	MRSA	GC inhibition at 25 μg/mL	[39]
				53 ± 15 (Ach@SeNP) 120 ± 23 (Qu–Ach@SeNP)	MDR *E. coli*	GC inhibition at 25 μg/mL	
	Na_2_SeO_3_	HSCH_2_CH_2_CO_2_H	Chitosan	Not reported	MRSA	ZOI: 0–4 nm	[40]
	Na_2_SeO_3_	C_6_H_8_O_6_	Polysorbate/Lysozyme	84	*E. coli* *S. aureus*	Concentrations of 1, 5, and 10 μg/mL led to inhibition	[41]
Pulsed laser ablation in liquids (PLAL)	Bulk Se pellets (target)	N/A	N/A	144 ± 46	MDR *E. coli*	MIC: 2.35 ppm	[42]
					MRSA	MIC: 14.25 ppm	
Laser ablation	Selenium plate	N/A	N/A	17	*E. coli*	ZOI: 55–69 mm	[43]
					*P. aeruginosa*	ZOI: 56–64 mm	
					*S. aureus*	ZOI: 33–54 mm	
					*B. subtilis*	ZOI: 38–44 mm	

**Abbreviations:** Polyvinyl acetate (PVA); ε-Poly-l-lysine (ε-PL); Sodium thiosulfate (Na_2_S_2_O_3_); L-ascorbic acid (C_6_H_8_O_6_); Bovine serum albumin (BSA); Carboxymethylcellulose (CMC); 3-Mercaptopropionic acid (HSCH_2_CH_2_CO_2_H); Methicillin-resistant Staphylococcus aureus (MRSA); Methicillin-resistant Staphylococcus epidermitis (MRSE); Minimum inhibitory concentration (MIC); Growth culture (GC); Zone of inhibition (ZOI).

**Table 2:** Advances of green synthesis of SeNPs with antimicrobial applications.

Methods	Species	NP Size (nm)	Tested Bacteria	Antibacterial Parameters	Ref.
Bacteria	*Lactobacillus Acidophilus*	50–80	*E. coli*	MIC: 9.4 ug/mL	[57]
			*S. aureus*	MIC: 1.2 ug/mL	
			*B. subtilis*	MIC: 3.5 ug/mL	
			*P. aeruginosa*	MIC: 6.5 ug/mL	
			*K. pneumoniae*	MIC: 4 ug/mL	
	*Streptomyces* sp.	20–150	*S. aureus*	MIC: 80–120 ug/mL	[56]
			*Acinetobacter* sp.	Synergistic ZOI range with different antibiotics: 5–30 mm	
			*B. subtilis*		
			*P. aeruginosa*		
			*K. pneumoniae*		
			*E. coli*		
	*Enterococcus faecalis*	29–195	*S. aureus*	ZOI: 8 mm	[55]
	*Ralstonia eutropha*	40–120	*E. coli*	MIC: 125 ug/mL	[47]
			*P. aeruginosa*	MIC: 100 ug/mL	
			*S. aureus*	MIC: 100 ug/mL	
			*S. pyogenes*	MIC: 250 ug/mL	
	*B. mycoides*	161	*P. aeruginosa*	MIC: 128 ug/mL	[54]
	*S. maltophilia*	171			
	*S. maltophilia*	221	*E. coli*	MIC: 125 ug/mL	[58]
			*P. aeruginosa*	MIC: 250 ug/mL	
			*S. aureus*	MIC: 250 ug/mL	
	*Bacillus amyloliquefaciens*	45–69	*S. aureus*	ZOI: 18.6 mm	[75]
			*B. subtilis*	ZOI: 6.3 mm	
	*Bacillus mycoides*	102–220	*S. aureus*	MIC: 78–156 ug/mL	[60]
			*P. aeruginosa*	MIC: 78–156 ug/mL	
	*Bacillus pumilus*	80–220	*S. aureus*	Biofilm formation: 40% decrease at 2 ug/mL	[76]
			*P. aeruginosa*		
	*S. aureus*	120–180	*S. aureus*	MIC: 75–150 ug/mL	[59]
	MRSA		*MRSA*		
	*E. coli*		*E. coli*		
	*P. aeruginosa*		*P. aeruginosa*		
Yeast and fungus	*S. cerevis*	30–100	*E. coli*	MIC: 31.25 ug/mL	[62]
			*P. aeruginosa*	MIC: 125 ug/mL	
			*K. pneumoniae*	MIC: 250 ug/mL	
			*S. aureus*	MIC: 31.25 ug/mL	
			*B. subtilis*	MIC: 250 ug/mL	
	*Penicillium chrysogenum*	12–84	*S. aureus*	ZOI: 20 mm	[63]
			*P. aeruginosa*	ZOI: 19 mm	
			*E. coli*	ZOI: 23 mm	
	*Penicillium corylophilum*	29–49	*S. aureus*	MIC: 9.37 ug/mL	[64]
			*B. subtilis*	MIC: 18.75 ug/mL	
			*E. coli*	MIC: 37.5 ug/mL	
			*P. aeruginosa*	MIC: 37.5 ug/mL	
	*Aspergillus Oryzae*	55–76	*K. pneumoniae*	ZOI: 13.6 mm	[65]
			*A. calcoaceticus*	ZOI: 15 mm	
			*E. cloacae*	ZOI: 14 mm	
			*E. agglomerans*	ZOI: 12.3 mm	
			*E. coli*	ZOI: 12.3 mm	
			*C. freundii*	ZOI: 12.6 mm	
			*P. mirabilis*	ZOI: 14.0 mm	
			*P. aeruginosa*	ZOI: 12.3 mm	
			*P. fluorescens*	ZOI: 11.6 mm	
			*MRSA*	ZOI: 16.6 mm	
			*E. faecalis*	ZOI: 13.0 mm	
			*E. feacium*	ZOI: 14.3 mm	
Plants	*Leucas lavandulifolia*	56–75	*E. coli*	ZOI: 15.33 mm	[68]
			*S. aureus*	ZOI: 13.33 mm	
			*S. epidermis*	ZOI: 15.33 mm	
			*S. typhi*	ZOI: 12.66 mm	
	*Withania somnifera*	45–90	*S. aureus*	ZOI: 19.66 mm	[69]
			*B. subtilis*	ZOI: 12 mm	
			*K. pneumoniae*	ZOI: 14 mm	
	*Zingiber officinale*	100–150	*Proteus* sp.	ZOI: 20 mm	[70]
			*Serratis* sp.	ZOI: 17 mm	
			*B. subtilis*	ZOI: 7 mm	
			*S. aureus*	ZOI: 10 mm	
			*K. pneumoniae*	ZOI: 3 mm	
			*E. coli*	ZOI: 13 mm	
	*Azadirachta indica*	142–168	*S. aureus*	ZOI: 14 mm	[71]
			*P. aeruginosa*	ZOI: 17 mm	
			*P. vulgaris*	ZOI: 15 mm	
			*B. cereus*	ZOI: 11 mm	
	*Spirulina platensis*	79 ± 44	*K. pneumoniae*	MIC: 25–250 ug/mL	[77]

**Abbreviations:** Minimal inhibitor concentration (MIC); zone of inhibition (ZOI); multidrug resistant (MDR); methicillin-resistant Staphylococcus aureus (MRSA).

## Data Availability

Not applicable.
